# The Book World of Medicine and Science

**Published:** 1901-08-24

**Authors:** 


### THE BOOK WORLD OF MEDICINE AND SCIENCE.

Tumours Innocent and Malignant, Their Clinical Features and Appropriate Tteatment. By J. Bland-Sutton. New Edition, with over three hundred engravings. (London: Cassell and Co. 1901. Price 21s.)

No one is better fitted to write a treatise on tumours than Mr. Bland-Sutton, accustomed as he is to deal with these abnormalities both from the pathological and the clinical points of view. In his classification of these diseases he begins by excluding from the tumours all those formations known as infective granulomata (a group which he says will no doubt be largely increased in the near future at the expense of the sarcomata, and in all probability of the carcinomata, as our knowledge of the biology of micro-organisms increases), and arranges the rest in four groups: 1. Connective-tissue tumours. 2. Epithelial tumours. 3. Dermoids. 4. Cysts. Each of these groups contains several genera, and each genus comprises one or more species. The classification adopted is throughout based on structure. The connective tissue tumours are thus divided into lipomata, chondromata, osteomata, odontomes (tumours arising from the germs of teeth), fibromata and myxomata, myelomata (tumours composed of red bone-marrow), sarcomata (malignant tumours composed of embryonic connective tissue), neuromata (including tumours arising from the sheaths of nerves as well as those which contain nerve cells; and the gliomata), angeiomata, lymphangeiomata (comprising endothelioma as well as the lymphatic nævi and cysts), and myomata. The epithelial tumours are somewhat more roughly divided into papillomata, adenomata, and carcinomata; but of course each of these genera is divided in turn into different species according to the particular tissue represented. Running across this structural classification is the other broad clinical division into innocent and malignant, examples of each of which are to be found in each of the first two groups. Working on this classification Mr. Bland-Sutton gives a full and very careful account of the different kinds of tumour met with in man, illustrating his points by frequent reference to comparative pathology. Interest at present centres so greatly in cancer that we naturally turn to the chapters dealing with that group of diseases. Mr. Bland-Sutton is careful not to adopt any of the various theories as to the causation of the disease, and as to remedies he can but urge removal as the only reasonable treatment. But removal must be early and complete, and some very important observations are made as to the necessity of so ordering the operative proceedings as to cut wide of the disease. "A striking feature of a cancer is that it is not circumscribed. When examined clinically it is rarely possible to define the limits between the tumour and the surrounding tissues. . . . This illimitation of cancer constitutes one of the greatest difficulties in dealing with it surgically, for it is clear, if with the aid of a microscope there is difficulty in defining its limits, how uncertain the surgeon must be in determining its extent with only fingers and eyes to guide him during an operation." Little liable as cancer may be to infect others, the disease is infective enough to a patient's own tissue. "In removing the infected organ, the infected lymphatics and blood-vessels, stuffed with the cancerous material, are divided, and the cancer cells are let loose over the damaged tissues, which they infect and lead to an extensive outbreak of local cancer." Hence the supreme necessity of exercising every care "not to incise the diseased part and thus unwittingly scatter the diseased cells over the denuded surfaces." In regard to cancer of the uterus, although the only hope lies in "the rough method" of complete removal, yet it is a melancholy fact that in cancer of the cervix "operative interference can only be carried out in a very small proportion of the cases with any prospect of success." The disease soon overruns the cervix and the propinquity of the bladder, rectum, etc., renders wide removal impracticable. "Look at vaginal hysterectomy for cancer from any point of view and the results can only be regarded as depressing." In cancer of the body of the uterus the results are better, and the best of all have followed abdominal hysterectomy for cancer of the body of the uterus. "The worst method of all is to dilate the cevical canal to be quite sure that the disease is cancer, and then perform vaginal hysterectomy." The chapters on dermoids are full of curious information, and indeed the book from beginning to end is one which may be read with interest and advantage. The illustrations are illustrative besides being in many cases pictorially very good. Some of them we think we have seen before.

Golden Rules of Aural and Nasal Practice. By Philip R. W. de Santi, F.R.C.S. "Golden Rules" Series, No. IX. (Bristol: John Wright and Co. Price 1s.)

To insist on the obvious sometimes verges upon the ridiculous. This is a teeny-weeny book containing eighty of the smallest possible pages. And although it is none the worse for that the author hardly need have taken the trouble to say in his preface that "no attempt has been made in this small book" to deal with the matter "other than briefly." The book, however, is a good one, and the short rules are well arranged to draw attention to the salient points in aural and nasal work. The continued appearance of number after number of this Golden Rules series shows, we suppose, that they meet a want. Clearly such small books can be of but little educational value, but they may be of great value as reminders, and as they are so small as to go quite easily into one's bag or even one's waistcoat pocket, their perusal no doubt serves well to occupy the many spare moments which occur even in the busiest lives.

Food Inspector's Handbook. By Francis Vacher, Medical Officer of Health for Cheshire. Third Edition. Illustrated. (London: The Sanitary Publishing Company. 1900. Price 3s. 6d. net.)

This is a small book, but it contains a vast amount of information on a considerable range of subjects, all bearing on the important one of food inspection. After a chapter on the qualifications and the equipment required by a food inspector, a short sketch is given of the statutory powers under which the inspection of food is carried out, and then the different forms of food are successively dealt with. Beginning with meat, and going through poultry, fish, fruit, and vegetables, bread, flour, milk, butter and cheese, etc., sweet-stuffs, tea and coffee, the book ends with meat again in the form of polonies, black-puddings, brawn, etc., and under each heading not only are the proper appearances described, but those also which result from the various diseases or adulterations to which the articles are subject. It is a useful book, and the author's position and experience give it great authority.

The Self-Educator in Chemistry. By James Knight, M.A., B.Sc. Edited by John Adams, M.A., B.Sc. (London: Hodder and Stoughton. 1901. Price 2s. 6d.)

The object of this book is that it shall operate as a self-educator, and its author says that it "does not prepare students for the examinations of the Science and Art Department or any other Department." Here then we have a teacher addressing his audience unfettered by any syllabus and untied by any attempt to get his audience through any examination, and we cannot but feel that his teaching gains in perspicuity by the freedom so obtained. Of course it is a popular book, and does not go very deeply into anything, but for those who wish to know something about chemistry and to study the matter for themselves by aid of experiments which they may perform in their own study, this will be found a very useful introduction to the science.

